# Science, Technology, Engineering and Mathematics (STEM) Comics as Cognitive Learning Enhancers for Malaysians

**DOI:** 10.21315/mjms2019.26.5.16

**Published:** 2019-11-04

**Authors:** Inderjit Kaur

**Affiliations:** Faculty of Science and Technology, Universiti Kebangsaan Malaysia, Bangi, Selangor, Malaysia

Dear Editor,

I read with interest the manuscript titled ‘Expanding opportunities for science, technology, engineering and mathematics subjects teaching and learning: connecting through comics’ by Adnan et al. (1).

It is indeed a very plausible article highlighting an interesting approach of making science, technology, engineering and mathematics (STEM) learning among Malaysian primary students more interesting. The approach of using STEM comics as proposed by this paper is an important innovation in the way STEM subjects are taught in Malaysian schools. This approach could instill an early interest as shown by this study for the primary school students to be involved in STEM fields. It is still an issue in Malaysia where students are afraid of choosing the science stream in their secondary school (2).

The approach proposed by this study could be an encouraging factor in instilling interest of students into the science field at a very young age. Comic strips have been proven by previous studies to be an effective tool to create interesting representation of teaching materials even from teachers with no background in comic designing (3).

Thus, the approach being proposed by this study is indeed practical and has high potential to be implemented in Malaysian primary schools by enhancing education via cognitive neurosciences.
